# Ambient Pressure Tympanometry Wave Patterns in Patients With Superior Semicircular Canal Dehiscence

**DOI:** 10.3389/fneur.2020.00379

**Published:** 2020-05-28

**Authors:** Anthony Thai, Zahra N. Sayyid, Davood K. Hosseini, Austin Swanson, Yifei Ma, Ksenia A. Aaron, Yona Vaisbuch

**Affiliations:** ^1^Department of Otolaryngology- Head and Neck Surgery, Stanford University School of Medicine, Stanford, CA, United States; ^2^Otolaryngology Head and Neck Department, Rambam Medical Center, Haifa, Israel

**Keywords:** ambient pressure tympanometry, superior semicircular canal dehiscence, vertigo, pulsatile tinnitus, autophony, hearing loss, temporal bone CT scan, vestibular evoked myogenic potentials

## Abstract

**Importance:** Superior semicircular canal dehiscence (SSCD) is a treatable condition, but current diagnostic modalities have numerous limitations. Clinicians would benefit from an additional tool for diagnostic workup that is both rapid and widely available.

**Objective:** To assess the utility of ambient pressure tympanometry (APT) in the diagnostic workup of SSCD by determining the sensitivity and specificity of APT for SSCD in comparison to other diagnostic modalities.

**Design:** Retrospective cohort study of patients who underwent APT and temporal bone computerized tomography (CT) scans from May 2017 to July 2018.

**Setting:** Tertiary referral center.

**Participants:** APT was performed as part of routine audiological testing on adult patients. We retrospectively analyzed all patients who received both APT and temporal bone CT scans, and divided ears into SSCD and non-SSCD groups based on the presence or absence of radiographic SSCD. Ears with other radiographic findings that could affect tympanic membrane compliance were excluded.

**Exposures:** All patients in this study underwent APT and temporal bone CT scans. Some patients also underwent pure tone audiometry and vestibular evoked myogenic potentials (VEMPs).

**Main Outcomes and Measures:** The primary outcome measures were sensitivity, specificity, and risk ratio of APT for SSCD. Secondary outcome measures include sensitivity of VEMPs and supranormal hearing thresholds.

**Results:** We describe 52 patients (70 ears) who underwent APT and CT imaging (mean age 47.1 years, 67.1% female). APT detected SSCD with 66.7% sensitivity and 72.1% specificity. In symptomatic patients, sensitivity was 71.4% and specificity was 75%. VEMPs performed best at detecting SSCD when defining a positive test as oVEMP amplitude >17 μV, with a sensitivity of 68.2%, similar to APT (*p* > 0.99). The combination of APT and VEMPs increased sensitivity to 88.9%, better than APT alone (*p* = 0.031) and trending toward better than VEMPs alone (*p* = 0.063).

**Conclusions and Relevance:** Rhythmic wave patterns on APT are associated with SSCD and may raise suspicion for this condition in conjunction with consistent results on other diagnostic modalities. Although clinical utility requires confirmation in a larger prospective study, APT is a simple, rapid, and widely available tool warranting further study.

## Introduction

Superior semicircular canal dehiscence (SSCD) was first described by Minor et al. in 1998 (1). Microscopic SSCD is found in 0.5% of temporal bone specimens (2) and 2–9% of temporal bone computed tomography (CT) scans depending on imaging technique (3–7). Due to a third mobile window effect, patients can present with vestibular and auditory symptoms, including autophony, aural fullness, sound-induced vertigo, pulsatile tinnitus, and hearing loss (1, 8–10). Surgical intervention provides partial or complete symptom resolution in up to 70% of patients (11–15). However, diagnosis is complicated by the variable presentation of SSCD, which may resemble otosclerosis and Meniere's disease (16, 17). Currently, CT imaging is required for diagnosis, but is not always feasible for initial workup due to cost, radiation exposure and limited access in some healthcare settings. Instead, the initial diagnostic algorithm in symptomatic patients involves vestibular examination and audiometry followed by vestibular evoked myogenic potentials (VEMPs) for diagnostic confirmation.

This initial workup has numerous limitations. In particular, it remains controversial which thresholds should be employed during audiologic and vestibular testing. On audiometry, SSCD patients may display low frequency air-bone gaps and supranormal bone conduction thresholds (SNT) above 0 dB (9, 18, 19). On VEMP testing, clinicians rely on abnormally low thresholds or high amplitudes, but precise cut-off values for either parameter remain uncertain (10). A recent study suggested that an ocular VEMP amplitude cutoff of 17 μV displays 100% sensitivity and specificity; although promising, these data have yet to be validated in other studies (20). Moreover, there are discrepancies between self-reported symptoms and imaging findings, poor correlation between vestibular testing and audiometry thresholds, and high false positive rates on CT imaging when compared to cadaveric studies (3–5, 21, 22).

Ambient pressure tympanometry (APT) uses a microphone to record changes in sound intensity in the external ear canal during introduction of a tone. Unlike standard tympanometry, the recording occurs over 15–20 s without alterations in external pressure. This allows for measurement of changes in external ear canal volume over time and indirect detection of tympanic membrane (TM) movement. A positive APT test consists of regular oscillations reflecting repeated TM fluctuations. Clinically, APT is solely employed in the workup of Patulous Eustachian Tube (PET); respiration-synchronous compliance changes have been reported in up to 75% of these patients (23–25). Compliance changes on APT have also been associated in small case series with glomus tumor, myoclonus, jugular bulb dehiscence, carotid artery dehiscence, and SSCD (26–30). Here, we present the first systematic analysis of the association between rhythmic APT wave patterns and SSCD, motivating further study of the utility of APT in the diagnostic workup of SSCD.

## Materials and Methods

### Patients

This study was approved by the Institutional Review Board (IRB-43715). From May 2017 to July 2018, APT was incorporated into routine audiologic testing, and was performed when possible on patients without specific indications or contraindications. Ears with sub-millimeter resolution temporal bone CT imaging were analyzed and divided into SSCD and non-SSCD groups based on the presence or absence of radiographic SSCD as determined by blinded imaging review by a neurotologist. Temporal bone CT scans consisted of images in the coronal, Stenvers view and Poschl views, all with slice thickness of 0.4 mm and maximum collimation of 0.625 mm. Some ears underwent APT twice during our study period. In cases where two tests showed one positive and one negative finding, we analyzed the test displaying rhythmic waves.

### Exclusion Criteria

We excluded patients with otologic diagnoses other than SSCD that might generate TM movement, including tegmen dehiscence, encephalocele, cholesteatoma, glomus tumors, jugular bulb dehiscence, sigmoid sinus diverticulum or dehiscence, aberrant carotid artery, carotid artery dehiscence, persistent stapedial artery, posterior semicircular canal dehiscence, middle ear myoclonus, and PET. Similarly, we also excluded patients with otologic conditions that might impair TM compliance, including otosclerosis, middle ear effusion, ossicular chain discontinuity, Meniere's disease, TM perforation and presence of pressure equalization tubes.

### Audiological Testing

All audiologic measurements were performed by trained audiologists in double-wall audiometric sound booths. APT was completed using Interacoustics Titan (Interacoustics, Audiometer Allé DK 5500 Middelfart) impedance devices controlled using the Titan Suite software v3.4. The software protocol used a 226 Hz probe tone presented at 85 dB to record ipsilaterally for 20 seconds with the instrument's air pump deactivated. The patient remained upright, seated, and quiet throughout the procedure. Hearing evaluations were completed using conventional audiologic procedures. The minimum battery included pure tone air and bone conduction audiometry, speech reception thresholds, and word recognition. Standard tympanometry and ipsilateral acoustic reflex testing were also completed.

VEMP testing was completed using an Intelligent Hearing Systems Smart USB (Intelligent Hearing Systems, 6860 SW 81st Street. Miami, FL 33143. USA) evoked potential system. Cervical (cVEMP) and ocular (oVEMP) VEMP threshold search procedures were completed for each ear. Air conduction 500 Hz tone bursts were used as stimuli. Ipsilateral cVEMP results were obtained with the patient reclined to 30 degrees above horizontal, with the head rotated 45 degrees from the test ear, and held above the exam Table 1 throughout each run. Contralateral oVEMP recordings were obtained with the patient seated upright with gaze 30 degrees above horizontal. Initial stimulus intensity was 105 dB presented via insert earphones and decreased in 10 dB steps until threshold was obtained. The stimulus rise, plateau and fall were 2, 1, and 2 ms, respectively. The highest intensity inter-amplitude was used for symmetry calculation.

**Table 1:** Diagnostic test characteristics.

	**SSCD Group**			**Symptomatic SSCD**		
	**Sensitivity** **(*N*)**	**Specificity** **(*N*)**	**Relative risk,** **95% CI,** ***p*-value**	**Sensitivity** **(*N*)**	**Specificity** **(*N*)**	**Relative risk,** **95% CI,** ***p*-value**
APT	66.7% (27)	72.1% (43)	2.67, 1.540 to 5.08, *p* = 0.003	71.4% (21)	75% (12)	2.08, 1.08 to 4.00, *p* = 0.028
cVEMP, thresh < 85 dB (500 Hz)	55.0% (20)	–	–	52.9% (17)	–	–
oVEMP, thresh < 85 dB (500 Hz)	50.0% (20)	–	–	52.9% (17)	–	–
oVEMP amp > 17 μV (500 Hz)	68.2% (22)	–	–	77.8% (18)	–	–
oVEMP, amp > 0 μV (4 kHz)	42.9% (14)	–	–	46.2% (13)	–	–
SNT	50.0% (22)	85.7% (42)	2.77, 1.48 to 5.17, *p* = 0.001,	62.5% (16)	91.7% (12)	2.58, 1.32 to 5.03, *p* = 0.006
APT or, oVEMP amp > 17 μV (500 Hz)	88.9% (27)	–	–	94.4% (18)	–	–

*N, total number; SSCD, superior semicircular canal dehiscence; APT, ambient pressure tympanometry; VEMP, vestibular evoked myogenic potential; oVEMP, ocular VEMP; cVEMP, cervical VEMP; amp, amplitude; thresh, threshold; SNT, supranormal threshold; CI, confidence interval*.

### Evaluation of APT Waves

Two authors performed independent, blinded review of APT waves and categorized them as rhythmic or noise. Rhythmic waves consisted of regularly spaced peaks with a frequency of 50–100 peaks per minute, consistent with physiologic heart rate. Noise consisted of fluctuations with no discernible peaks, inconsistently spaced peaks, or a frequency outside of 50–100 peaks per minute. In cases of inconsistent classification of APT tracings between reviewers, the authors came to an agreement following discussion while still blinded to patient diagnosis. For each ear, amplitude and frequency were calculated using a novel algorithm created in RStudio (v1.1.463, RStudio, Inc., Boston, MA, USA). We defined the wave amplitude for each ear as the average height of the waveforms (measured from peak to trough) present throughout the 20 s recording. To account for noise, all heights greater than two standard deviations away from the mean amplitude were discarded as outliers. Frequency was determined by quantifying the number of measured peaks per minute.

### Evaluation of Symptoms and Vestibular and Audiometric Tests

Ears were analyzed regarding presence of SNT on audiometry, abnormal VEMPs, and SSCD symptoms. Patients were considered symptomatic if they reported at least one of the following symptoms: pulsatile tinnitus, autophony, ear fullness, sound-induced vertigo. SNT was defined as a bone conduction threshold better than 0 decibels (dB) on pure tone audiometry. Based on previous literature (20) and guidelines at our institution, several definitions were employed for positive VEMP findings: cVEMP threshold at 500 Hz below 85 dB, oVEMP threshold at 500 Hz below 85 dB, oVEMP amplitude at 500 Hz greater than 17 μV, or oVEMP amplitude at 4 kHz greater than 0 μV. Sensitivity for radiographic SSCD was calculated for each of these cutoffs.

### Statistical Analysis

The chi-square test was used for inter-group comparison of patient sex. Student's *t*-test was used for inter-group comparison of patient age as well as frequency and amplitude on APT tracings. To examine associations between diagnostic tools and CT results, relative risk and 95% confidence interval were estimated by employing a Poisson regression model with robust sandwich variance estimator that corrects for potentially overestimated standard error (31). Sensitivity and specificity were calculated based on 2 x 2 frequency tables of diagnostic tools and CT results. The McNemar chi-square test was used to compare sensitivity and specificity values between diagnostic tools (32). All analyses were performed using SAS software version 9.4 (SAS Institute, Cary, NC).

## Results

### Patients

A total of 469 patients (780 ears) underwent APT testing. From this sample, 89 patients (168 ears) underwent temporal bone CT imaging. 98 ears were excluded due to the following diagnoses: tegmen dehiscence (23), otosclerosis (16), middle ear effusion (11), sigmoid dehiscence (9), glomus tumor (6), cholesteatoma (6), myoclonus (4), TM perforation (4), carotid artery dehiscence (4), PE tube placement (4), jugular bulb dehiscence (3), encephalocele (2), and one each of ossicular chain discontinuity, Eustachian tube dysfunction, Meniere's disease, posterior semicircular canal dehiscence, sigmoid diverticulum, and aberrant carotid artery. The study cohort included 52 patients (70 ears) with mean age 47.1 ± 16.7 years and consisting of 47 ears from female patients (67.1%). Based on radiographic findings, ears were divided into SSCD (27 ears) and non-SSCD groups (43 ears). These groups were similar in sex (non-SSCD: 65.1% female, SSCD: 70.4% female, *p* =.649) and age (non-SSCD: 46.7 ± 17.2 years, SSCD: 47.9 ± 16.3 years, *p* = 0.777).

Four ears in each group underwent APT twice during the study period. In the SSCD group, 3 of these 4 ears had inconsistent results between tests (one rhythmic wave and one with noise), compared to 1 of 4 ears in the non-SSCD group.

### APT Outcomes

**Figure 1:**
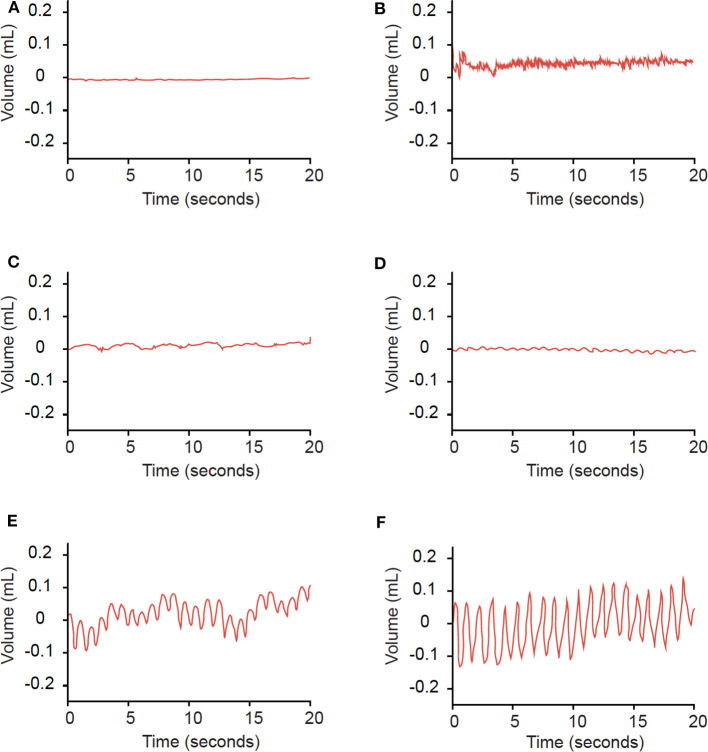
Examples of findings on APT. Findings of a horizontal line **(A)**, no regularly spaced peaks **(B)**, or frequencies outside of 50–100 peaks/minute **(C)** were categorized as noise. Waves with consistently-spaced peaks and frequencies of 50−100 peaks/minute **(D–F)** were categorized as rhythmic waves.

Examples of rhythmic waves and noise are displayed in Figure 1. In detecting radiographic SSCD, rhythmic APT waves displayed 66.7% sensitivity (27 SSCD ears) and 72.1% specificity (43 non-SSCD ears) (Table 1). The relative risk of radiographic SSCD in ears with rhythmic waves compared to noise was 2.67 (*p* = 0.003). The average amplitude in the SSCD group (0.03 mL) was significantly greater than that of the non-SSCD group (0.015 mL, *p* = 0.01). In symptomatic ears, rhythmic APT waves displayed 71.4% sensitivity (21 SSCD ears) and 75% specificity (12 non-SSCD ears, relative risk 2.08).

In this study, we excluded 16 SSCD ears with comorbid otologic pathologies that may cause TM oscillations. The most common comorbidity was tegmen dehiscence (10 ears, 23.3% of SSCD ears). The sensitivity of APT in these 16 SSCD ears with additional pathology was 50%.

### Comparison of APT Results to Other SSCD Screening Tools

In this study cohort, 81.5% of SSCD ears and 23.3% of non-SSCD ears underwent VEMP testing. Due to the small sample of non-SSCD ears undergoing VEMP testing, we do not report the specificity of VEMPs in this study. With regards to sensitivity, several definitions of positive VEMP findings were employed. Of these definitions, oVEMP amplitude > 17 μV at 500 Hz had the highest sensitivity for radiographic SSCD (68.2%), performing similarly to APT (*p* > 0.99). APT also performed similarly to SNT with regards to sensitivity (*p* = 0.125) and specificity (*p* = 0.30). Importantly, the presence of either a positive APT finding or an oVEMP amplitude > 17 μV displayed 88.9% sensitivity, better than APT alone (*p* = 0.031) and trending toward better than oVEMP amplitude > 17 μV alone (*p* = 0.063). However, only 40.9% of ears with radiographic SSCD displayed both rhythmic APT waves and oVEMP amplitude > 17 μV.

In symptomatic patients, oVEMP amplitude > 17 μV displayed 77.8% sensitivity for radiographic SSCD, performing similarly to APT (*p* = 0.727). APT performed similarly to SNT in this subgroup (*p* = 0.375). The presence of either oVEMP amplitude > 17 μV or a rhythmic APT wave displayed 95% sensitivity for radiographic SSCD, higher than VEMPs alone (*p* = 0.025) and trending toward higher than APT alone (*p* = 0.063). 50% of symptomatic SSCD patients displayed both rhythmic APT waves and oVEMP amplitude > 17 μV.

## Discussion

Although SSCD is treatable, its diagnosis presents a clinical challenge. CT scans are required for SSCD diagnosis but are time-consuming, not universally available, and associated with a risk of radiation exposure (33). We introduce APT as a simple, rapid and widely available tool that may display rhythmic waves in SSCD patients. To test this association, we rely on CT imaging for confirmation of SSCD diagnosis, as most patients in our cohort lack surgical confirmation. Our initial data suggest an association between rhythmic APT waves and SSCD, particularly in symptomatic patients. Pending validation in a larger prospective study, APT may be a useful addition to the workup of SSCD in conjunction with current diagnostic tools.

The 43 SSCD ears in this study (27 ears with SSCD only and 16 SSCD ears with comorbid pathology) were briefly described in a case series, which did not include a control group or associated data on symptoms, audiometry and vestibular testing (30). To our knowledge, these two studies are the only systematic studies in the English literature that evaluate APT in the workup of conditions other than PET.

**Figure 2:**
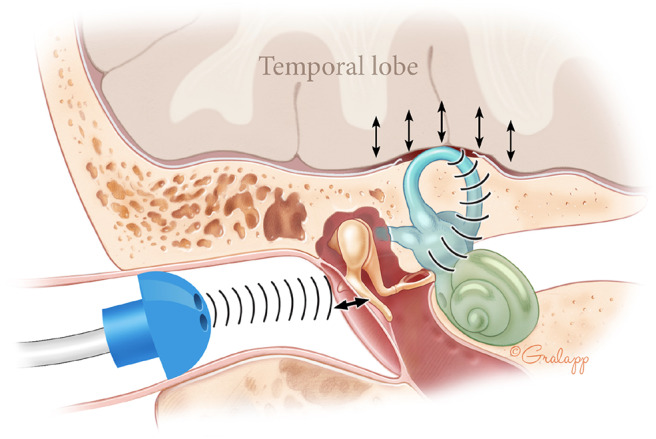
Theory for presence of APT waves in SSCD patients. In SSCD, the brain pounds rhythmically on the dehiscent superior canal. These oscillations propagate sequentially through inner ear fluids, the oval window, and the ossicular chain. This generates rhythmic TM oscillation that may be detected as rhythmic waves on APT.

APT passively records external ear canal volume over time in resting patients, a proxy for TM movement. In healthy ears, the TM should not move appreciably over this timescale. However, dehiscence of the bony layer overlying the superior canal may allow transmission of sound pressure from cerebral vessels through this open window (28). These oscillations may propagate sequentially through inner ear fluids, the oval window, the ossicular chain, and the TM (Figure 2). Due to this hypothesis, we excluded ears with conditions that might affect TM movement from both SSCD and non-SSCD groups.

### APT Outcomes

In this study, APT detected radiographic SSCD with 66.7% sensitivity and 72.1% specificity (Table 1). In symptomatic patients, APT displayed sensitivity of 71.4% and specificity of 75%. SSCD workup would only be performed in these symptomatic patients, as no indication currently exists for treatment of asymptomatic SSCD. However, the small size of our cohort should prompt cautious interpretation. Moreover, in SSCD ears with additional pathology, the sensitivity of APT decreases to 50%. Pressure waves from multiple sources may interact and cause noisy TM fluctuation, limiting APT's utility in these patients.

For ears with contradictory results between two APT tests, we analyzed the test with the rhythmic wave, and applied this standard to SSCD and non-SSCD groups. Noise on APT suggests lack of pathology or high levels of noise obscuring existing pathology, while rhythmic waves indicate presence of a source for TM fluctuation. We hypothesize that SSCD ears display rhythmic waves on some but not all APT tests due to technical challenges that decrease the signal to noise ratio, including improper seal formation with the APT probe or excessive patient breathing or movement. In contrast, healthy ears should not occasionally produce rhythmic APT waves. Therefore, for ears with contradictory APT findings, we speculate that noise arises from technical issues and select rhythmic waves for analysis. The equal application of this standard to both groups may lead to increased sensitivity and decreased specificity. To limit noise, we recommend ensuring proper seal formation, instructing patients to limit heavy breathing and movements, and performing APT for longer periods to better detect existing pathology.

Aside from these technical challenges, we propose several reasons for the lack of rhythmic waves in some SSCD ears. CT scans may have a high false positive rate for SSCD (4, 5, 22). Therefore, patients in our SSCD group with negative APT findings may have intact but thin superior semicircular canals that appear radiographically dehiscent. In fact, patients with near-dehiscent superior canals can display SSCD symptoms, but may not have APT findings due to an intact barrier preventing transmission of sound pressure (34). Alternatively, small areas of dehiscence may not transmit waves of sufficient amplitude for detection, a hypothesis that cannot be confirmed due to the difficulty of measuring dehiscence area on CT imaging. Moreover, additional pathologies other than those in our exclusion criteria may limit TM mobility.

In addition, several factors may contribute to the presence of rhythmic waves in non-SSCD ears. Most importantly, wave patterns were categorized blindly but subjectively as rhythmic or noise. We may have employed a low threshold for categorizing a wave as rhythmic, leading to overestimation of sensitivity and underestimation of specificity. To address this problem, we have developed a preliminary algorithm to filter out baseline noise and more objectively identify rhythmic waves. This algorithm will need to be validate in a large, prospective sample, which is the focus of a future study. Secondly, APT may constitute an overly sensitive test and detect low amplitude oscillations in healthy ears. Consistent with this reasoning, the wave amplitude in the SSCD group (0.03 mL) was significantly larger than that in the non-SSCD group (0.015 mL, *p* = 0.001). Setting an amplitude threshold for a positive APT test may reduce the false positive rate but would decrease sensitivity. Lastly, other pathologies not considered in our exclusion criteria may cause TM fluctuation in our non-SSCD cohort.

### Comparison of APT Results to Other SSCD Screening Tools

In our patient cohort, defining a positive VEMP as oVEMP > 17 μV yielded the highest sensitivity (68.2%), performing similarly to APT (Table 1, *p* > 0.99). A previous study described higher VEMPs sensitivity for radiographic SSCD (91%) than reported in our study. This study defined a positive VEMP result as any VEMP threshold < 65 dB at 250, 500, or 1000 Hz (6). Our study evaluated VEMPs performed at 500 Hz; these different frequencies may partially account for the discrepant sensitivities. Another study of 29 patients with surgically confirmed SSCD determined that oVEMP amplitude > 17 μV at 500 Hz displayed a sensitivity of 100%, compared to 68.2% in our study (20). However, the above study performed analysis by patient, while our study analyzed SSCD by ear. SSCD patients undergoing surgery also likely displayed symptoms. When analyzing our symptomatic cohort by patient, sensitivity increased to 84.6% (13 patients). The small sample size and lack of surgical confirmation in our study may account for the remaining gap in sensitivity.

In detecting radiographic SSCD, APT performed similarly to SNT in sensitivity (*p* = 0.125) and specificity (*p* = 0.302). Moreover, APT increases sensitivity and specificity when combined with other SSCD screening tools. The presence of rhythmic APT waves or oVEMP amplitude > 17 μV displayed better sensitivity than APT alone (*p* = 0.031) and trended toward better sensitivity than oVEMP alone (*p* = 0.063). A subgroup of symptomatic patients displayed similar results (Table 1). Pending validation of these data, APT testing of symptomatic patients in resource-poor settings may inform whether patients should be recommended for CT imaging.

### Limitations

This is a small, single-center retrospective study without routine or randomized CT imaging. While APT was performed routinely, CT imaging was likely performed more frequently in patients with symptoms and/or test results raising suspicion for otologic pathology. With randomized imaging, fewer patients in each group might have symptoms, abnormal VEMPs, or SNT. Therefore, our study may have overestimated sensitivity and underestimated specificity, with an unclear bias on relative risk. In contrast, CT imaging may have high false positive rates for detecting radiographic SSCD (4, 5, 22), which may falsely increase sensitivity and reduce specificity. A prospective study is required to address selection bias and surgical confirmation is needed to correct for false positive rates of CT imaging.

APT is a simple, rapid, and widely available test. Preliminary results suggest that characteristic APT wave patterns may raise suspicion for SSCD in symptomatic patients, in conjunction with consistent results on other diagnostic modalities. These data motivate a prospective study to evaluate the utility of APT in the diagnostic workup of SSCD.

## Data Availability Statement

The datasets generated for this study are available on request to the corresponding author.

## Ethics Statement

The studies involving human participants were reviewed and approved by Stanford University Institutional Review Board (IRB-43715). Written informed consent for participation was not required for this study in accordance with the national legislation and the institutional requirements.

## Author Contributions

AT and ZS conducted data acquisition, analysis, and interpretation, and made significant contributions to the writing and editing of the manuscript. They are designated co-first authors for this study. DH was involved in project conception, manuscript editing and creation of figures. AS contributed to data acquisition, data analysis and manuscript review. YM played a significant role in the statistical analysis of the data and manuscript review. KA was involved in data interpretation and manuscript review. YV led the conception of the study, and made significant contributions to data interpretation and manuscript review.

## Conflict of Interest

The authors declare that the research was conducted in the absence of any commercial or financial relationships that could be construed as a potential conflict of interest. The reviewer CMA and handling editor declared their shared affiliation at the time of the review.

## Abbreviations

SSCD: superior semicircular canal dehiscence
APT: ambient pressure tympanometry
CT: computerized tomography
cVEMP: cervical vestibular evoked myogenic potential
dB: decibel
oVEMP: ocular vestibular evoked myogenic potential
PCHL: pseudo-conductive hearing loss
PE: pressure equalization
PET: patulous Eustachian tube
ROC: receiver operating characteristic
TM: tympanic membrane
VEMP: vestibular evoked myogenic potential.
